# Self-assembling Molecular Medicine for the Subacute Phase of Ischemic Stroke

**DOI:** 10.1007/s11064-022-03638-5

**Published:** 2022-06-06

**Authors:** Takahiro Muraoka, Itsuki Ajioka

**Affiliations:** 1 Department of Applied Chemistry, Graduate School of Engineering, Tokyo University of Agriculture and Technology, Tokyo, 184-8588 Japan; 2 Kanagawa Institute of Industrial Science and Technology (KISTEC), Kanagawa, 243-0435 Japan; 3 Center for Brain Integration Research (CBIR), Tokyo Medical and Dental University (TMDU), Tokyo, 113-8510 Japan

**Keywords:** Self-assembling molecular medicine, Ischemic stroke, Subacute phase, Regeneration, Artificial ECM, Supramolecular peptidic scaffolds

## Abstract

Ischemic stroke leads to acute neuron death and forms an injured core, triggering delayed cell death at the penumbra. The impaired brain functions after ischemic stroke are hardly recovered because of the limited regenerative properties. However, recent rodent intervention studies manipulating the extracellular environments at the subacute phase shed new light on the regenerative potency of the injured brain. This review introduces the rational design of artificial extracellular matrix (ECM) mimics using supramolecular peptidic scaffolds, which self-assemble via non-covalent bonds and form hydrogels. The facile customizability of the peptide structures allows tuning the hydrogels' physical and biochemical properties, such as charge states, hydrophobicity, cell adhesiveness, stiffness, and stimuli responses. Supramolecular peptidic materials can create safer and more economical drugs than polymer materials and cell transplantation. We also discuss the importance of activating developmental programs for the recovery at the subacute phase of ischemic stroke. Self-assembling molecular medicine mimicking the ECMs and activating developmental programs may stand as a new drug modality of regenerative medicine in various tissues.

## Introduction

Stroke is the second-leading cause of death in the world and remains the cause of severe disability [1]. The disability-adjusted life year (DALY) measures population health that accounts for both mortality and nonfatal disability proposed by the World Bank and the World Health Organization (WHO). The global burden of diseases, injuries, and risk factors study (GBD) reported 101 million prevalent cases of stroke and 143 million DALYs in 2019, which implicates 143 million-year loss of the world’s whole population due to stroke. In Japan, stroke is the fourth-leading cause of death following cancer, heart disease, and senility [2], and there were 1.11 million stroke patients in 2017 [3]. The direct and indirect cost of stroke was estimated as USD60 billion/year in Japan [4], higher than USD50 billion/year in the United States [5]. Thus, stroke has been a big public concern from a financial view and patients' quality of life.

Of all strokes, 87% are ischemic strokes caused by blood supply interruption, mainly from the cerebral artery [5]. Consequently, glutamate accumulates in the extracellular spaces and promotes neuron death, forming an injured core [6]. This excitatory cell death triggers delayed cell death caused by peri-infarct depolarizations and inflammation at the penumbra, the surrounding area of the ischemic core [7]. Thus, the number of neurons decreases over a few weeks after the ischemic stroke's onset. The impaired brain functions caused by ischemic stroke are generally not recovered because the mammalian central nervous system (CNS) has limited regenerative ability after injury. In the mammalian CNS, neurons are generated from neuronal stem/progenitor cells during only developmental stages except for the specific types of neurons generated at the adult stages from the ventricular-subventricular zone (V-SVZ) of the lateral ventricle and the dentate gyrus of the hippocampus [8]. Once neurons are generated from stem/progenitor cells and exit their cell cycle, they immediately lose their proliferative potency and do not produce replacing neurons [9]. In addition to the irreversible cell cycle exit, the plasticity of neuronal circuits decreases after a critical period in the early postnatal stages [10, 11]. Thus, the mammalian CNS is prone to lose the ability to rebuild neuronal circuits after the injury.

However, recent rodent intervention studies manipulating the extracellular environments shed new light on the injured brain's regenerative potency, especially at the subacute phase of ischemic stroke, around 1 week after the onset. In this review, we introduce a synthetic approach for mimicking the extracellular matrix (ECM) and discuss the possible mechanisms of injured brain regeneration by introducing the rodent intervention studies at the subacute phase. Finally, we propose self-assembling molecular medicine mimicking the ECMs as a new drug modality for regenerative medicine.

## Synthetic Approach to Developing ECM Mimics

The ECM is one of the primary factors for tissue development [12–15]. The ECM is an extensive macromolecular network mostly comprised of fibrillar and degradable proteins such as collagen, fibronectin, and laminin. The network of the fibrillar proteins provides physical scaffolding and mechanical stability for the resident cells and holds water for tissue hydration. The ECM plays a key role in regulating cell adhesion by binding to such as the cell surface receptors Integrins. Integrin regulates the cytoskeleton remodeling, thereby controlling cell survival, proliferation, and differentiation. In addition, growth factors are incorporated into and released from the ECMs to coordinate such multiple cellular events for tissue development, repair, and homeostasis. Such multiple functions of the ECMs inspired the design of biomaterials as ECM mimics to accelerate injured tissue regeneration [16, 17]. Although natural scaffold materials are available as ECM mimics, such as Matrigel, collagen, silk, and decellularized ECMs, they have potential difficulties in tuning the physicochemical properties and variances among lots. To construct synthetic ECM mimics, polymeric materials such as poly(ethylene glycol) and poly(vinyl alcohol) have been utilized [18, 19]. Although they are tunable in terms of chemical, biological and mechanical properties for specific functionalization and three-dimensional spatiotemporal patterning [20], the potential antigenicity could limit their in vivo applications [21]. In this context, synthetic hydrogels consisting of short peptides possess tremendous advantages owing to the established chemical synthesis enabling the design of peptides with diverse primary structures. The rational design of peptides allows for functional self-assembling structures with desirable properties and bioactivities. Mainly, one-dimensional self-assembly of the peptides provides nanofibers. The supramolecular peptidic nanofibers entangle to form a network, indicating morphological similarity to the native ECM. The facile customizability of the peptide structures allows tuning the hydrogels' physical and biochemical properties, such as charge states, hydrophobicity, cell adhesiveness, stiffness, and stimuli responses. Furthermore, the supramolecular systems, in which molecules self-assemble via non-covalent bonds, possess additional advantages such as a thixotropic property, allowing for becoming fluid when agitated and returning to a gel state instantly at rest, beneficial for injection and processability for attaching bioactive epitopes and proteins. These unsurpassed functionalities of the supramolecular peptidic hydrogels make them ideal ECM-mimic biomaterials to be explored for tissue engineering in vitro and injured tissue regeneration in vivo.

## Supramolecular Peptidic Scaffolds for Tissue Engineering

### Amphiphilic Peptides with Alternating Hydrophilic and Hydrophobic Side Chains

**Fig. 1:**
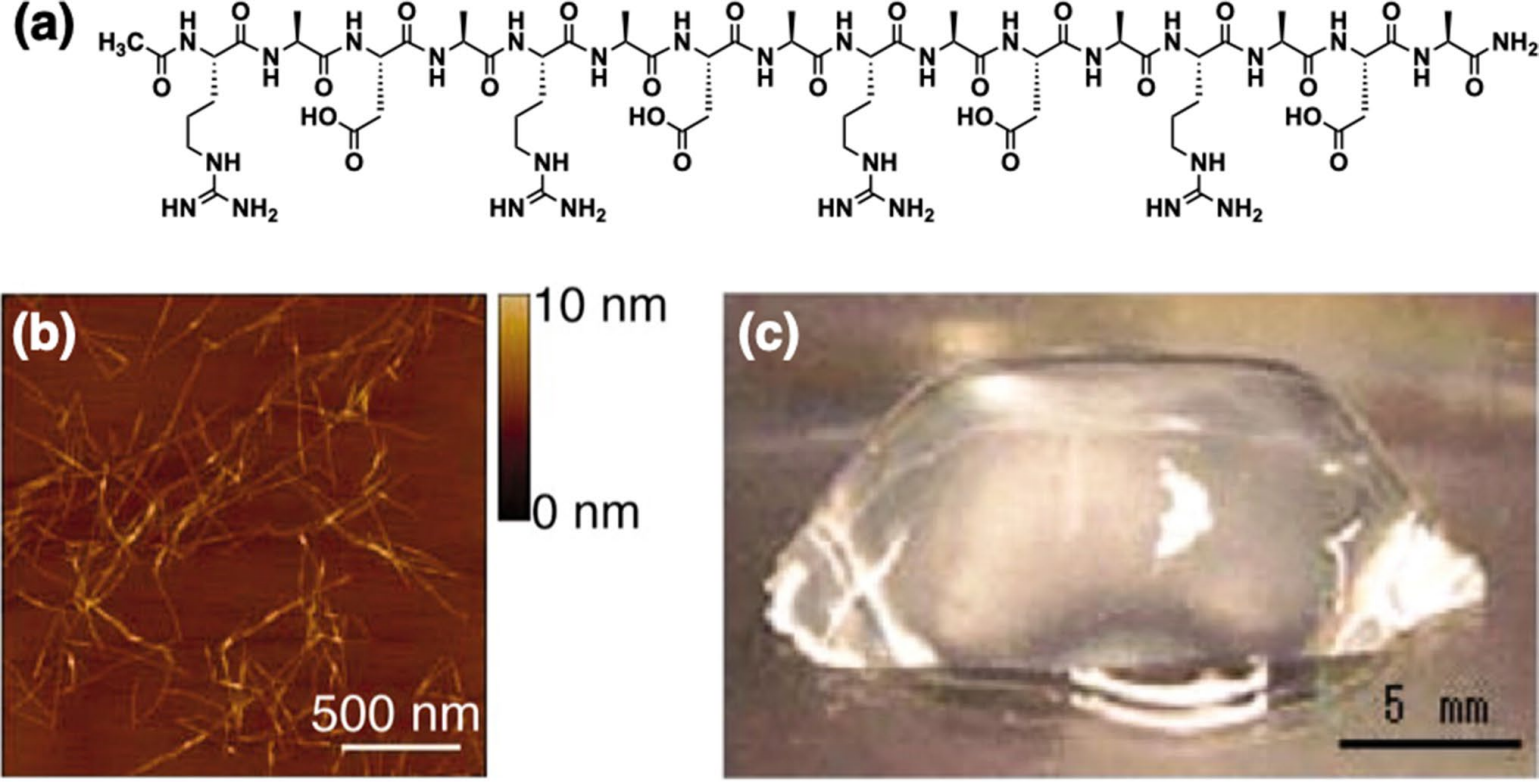
Self-assemblies of RADA16 peptides. **a** Molecular structure of RADA16. **b** AFM image of RADA16 nanofiber scaffold. **c** Photograph of RADA16 hydrogel at 0.5 wt%. Copyright 2005 National Academy of Sciences

A typical structure of fiber-forming peptides is an amino acid sequence with alternating hydrophilic and hydrophobic residues. (RADA)4, also called RADA16, developed by Zhang and coworkers, is a pioneering example of this class of self-assembling peptides (Fig. 1a) [22–24]. The peptides with this characteristic sequence expose a hydrophobic surface at one side of the molecules, suitable for self-assembly by hydrophobic interactions and hydrogen-bond interaction at the main chain to form a one-dimensional β-sheet assembly and a hydrogel (Fig. 1b, c). Importantly, RADA16-based nanofibers show a cell adhesive property, making RADA16 an attractive material for in vitro and in vivo. Koss and coworkers assessed the biocompatibility of RADA16-based scaffolds in brain tissues in vitro using primary cultured rat microglia and in vivo by intracerebral injection into newborn rats [25]. The cultured microglia on a poly-L-lysine (PLL) and a RADA16 scaffold were treated with lipopolysaccharide (LPS) to stimulate an inflammatory response. No significant differences were observed in the release of pro-inflammatory cytokines IL-1β and TNF-α and nitric oxide (NO) between the PLL and RADA16. For in vivo experiments, microglial activation was investigated by the immunostaining with the microglia marker CD68 antibody at the site of the intracerebral injection, where no significant increase in the number of activated microglia was detected compared to the saline treatment. Considering these results, RADA16 shows favorable biocompatibility in primary microglia culture in vitro and brain tissue in vivo. Based on the biocompatibility, functionalization of RADA16 with bioactive motifs has been demonstrated. A representative example is providing a laminin epitope, such as the IKVAV sequence [26] to RADA16. Cheng and coworkers injected RADA16-IKVAV encapsulating neural stem cells (NSCs) into the injured brain immediately after traumatic injury treatment and found enhanced survival of the encapsulated NSCs, a reduction of the glial activation, the support of the encapsulated NSCs in neuronal differentiation after six weeks of the transplantation compared to those in RADA16 [27].

**Fig. 2:**
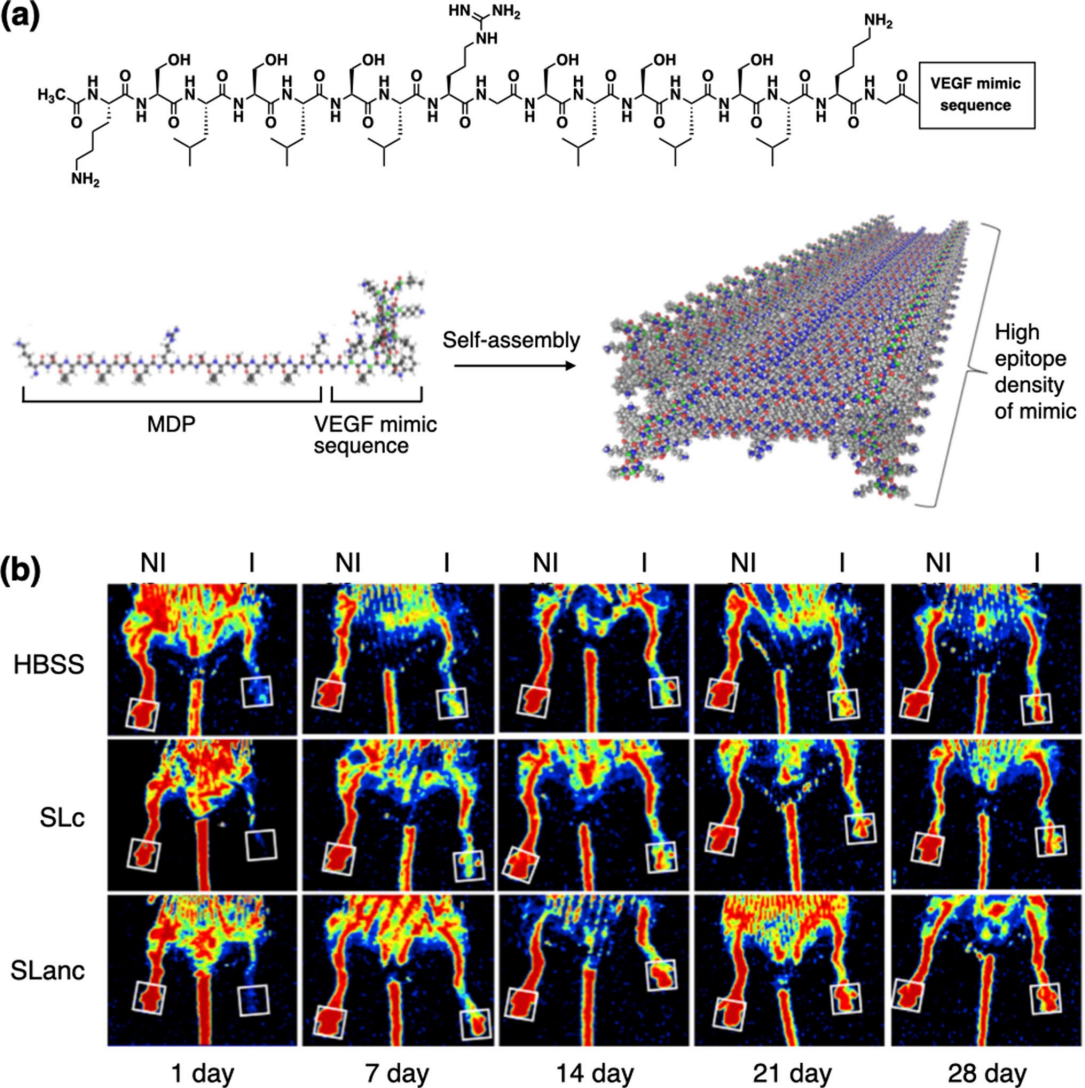
Self-assemblies of MDP peptides. **a** Molecular structure of MDP and a schematic image showing how the MDPs self-assemble into nanofibers through hydrophobic packing and hydrogen bonding. **b** Recovery from hind limb ischemia after treatment with SLanc (angiogenic peptide; MDP-VEGF mimics conjugate), SLc (base peptide; MDP), or HBSS (carrier control). Laser Doppler perfusion imaging (LDPI) showed rapid restoration of blood flow to the foot pad (boxed region) in SLanc-treated 13 month old mice, as compared to that in control SLc- and HBSS-treated mice. NI and I represent nonischemic and ischemic legs, respectively. Copyright 2016 Elsevier

Hartgerink and coworkers have developed multidomain peptides (MDPs), such as K2(QL)6K2, based on the alternating hydrophilic and hydrophobic amino acid residues at the core and the terminal charged amino acid residues [28]. Glutamic acid or lysine was used as the terminal residues to afford a net negative or positive charge, respectively. As the hydrophilic amino acids, cysteine, glutamine, serine, and threonine were used, while amino acids with aliphatic and aromatic side chains were used as the hydrophobic residues. By systematic screening, they have concluded that a proper balance of the charged residues to the repeating hydrophilic–hydrophobic residues is necessary for the supramolecular fiber formation maintaining solubility; a typical requirement is at least three times more the repeating residues than the charged residues [29]. The terminal charged residues serve two functions: first, to increase the solubility, and second, to cross-link the peptide fibers through non-covalent interactions. The addition of multivalent salts with charges opposite to those of the peptide termini allows cross-linking of the fibers to trigger hydrogelation [30]. Such supramolecular interactions are capable of reformation after disruption so that the MDP hydrogels are thixotropic. By incorporating a bioactive amino acid sequence at the terminus or the middle of the peptide, MDPs can be customized to initiate a specific cell response. For example, incorporating the RGD sequence, derived from an ECM protein fibronectin [31], at the C-terminus of an MDP can promote cell viability, spreading, and proliferation [32]. An MDP responsive to a cellular degradation signal was also developed, mimicking the cellular remodeling of the ECMs. Incorporation of the LRG sequence at the core of the MDP allowed for the peptide cleavage by matrix metalloprotease-2 (MMP-2). Cells grown on top of the MDP hydrogel migrated into the hydrogel only when the MDP contained the LRG sequence [33]. An MDP bearing vascular endothelial growth factor (VEGF) mimic sequence KLTWQELYQLKYKGI [34] has also been synthesized (Fig. 2a). This particular MDP can activate VEGF receptors to promote angiogenesis and recovery after hind limb ischemia (Fig. 2b) [35, 36].

### Peptide Amphiphiles with a Hydrophobic Alkyl Tail

**Fig. 3:**
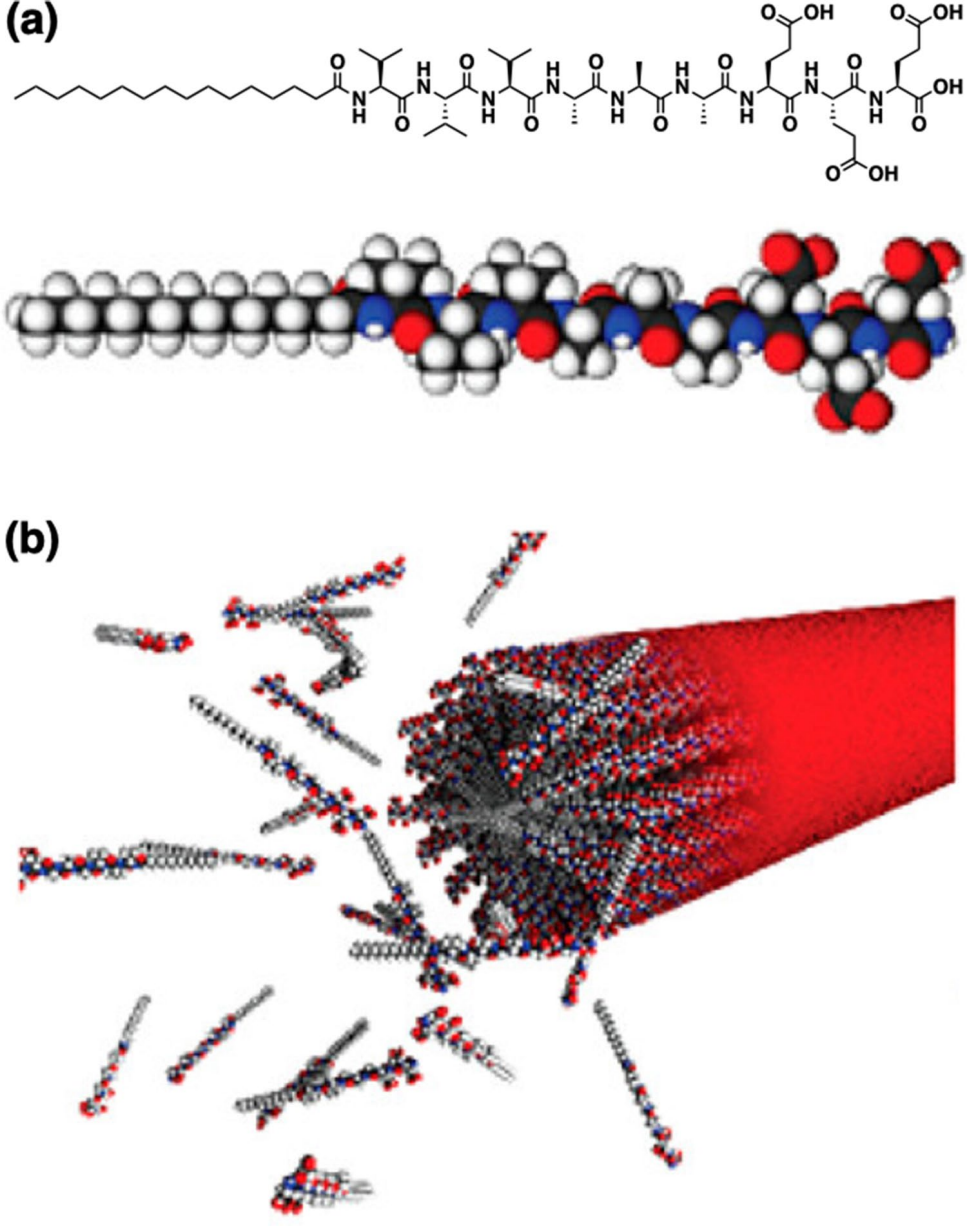
Self-assemblies of Pas. **a** Molecular structure of a peptide amphiphile (PA). **b** Schematic illustration of the self-assembling process of a PA forming a nanofiber. Copyright 2017 American Chemical Society

Lipidated peptides attached to an alkyl tail are an important family of peptide materials useful for ECM-mimetic cell scaffolds. In 2001, Stupp and coworkers reported peptide amphiphiles (PAs) self-assembled into nanofibers to form hydrogels [37, 38]. A typical molecular structure of PAs is a peptide containing fewer than ten amino acid residues conjugated with an alkyl tail longer than ten carbon atoms (Fig. 3a). The peptide segment consists of a domain with a propensity of the β-sheet formation adjacent to the alkyl tail and terminal residues with charges to increase water solubility. A representative self-assembling structure of PAs is cylindrical micelles affording nanofibrous superstructures with high aspect ratios by the hydrophobic collapse of the alkyl tails and hydrogen bonds at the peptide segment (Fig. 3b). The amino acid residue next to the alkyl tail plays a particularly important role in the nanofibrous formation [39], and putting a photocleavable steric unit at this amino acid residue enables light-triggered fiber formation, in turn, hydrogelation [40, 41]. Based on the well-defined self-assembling structures, it is possible to display bioactive components on the surface of the nanofibers by attaching them to an end of the PAs with flexible spacers. This bottom-up construction of bioactive nanofibers promotes neuronal differentiation of stem/progenitor cells in vitro using PAs presenting a laminin epitope IKVAV [42]. Hierarchical assemblies of PAs over macroscopic scales have also been demonstrated. Monodomain viscoelastic strings were developed over centimeter scales by annealing and dragging the PA liquid crystal from a pipet into salted media [43]. These PA strings were bioactive, in which the oriented growth of neurites was promoted [44]. Recently, the Stupp group reported promoting recovery from spinal cord injury by bioactive scaffolds integrating two different orthogonal epitopes. One signal can activate β1-integrin and a second one contributes to activating the fibroblast growth factor 2 (FGF2) receptor. By tuning the amino acid sequence near the alkyl tail, the motions of PA molecules within the scaffold fibrils could be modulated, which further led to significant influences on vascular growth, axonal regeneration, myelination, survival of motor neurons, reduced gliosis, and functional recovery after acute spinal cord injury in mice [45].

As the hydrophobic tail, drug molecules can be conjugated to the β-sheet-forming peptides [46]. Cui and coworkers have developed peptide-drug conjugates using anticancer drugs such as camptothecin (CPT), a DNA topoisomerase I inhibitor [47]. The CPT and peptide segments are connected by a reducible disulfylbutyrate linker. The CPT-peptide conjugate formed self-assemblies into nanofibers or nanotubes. In the presence of glutathione, a cancer-relevant reducing agent, faster release of the bioactive CPT was observed than in its absence, and in vitro efficacy against a number of cancer cell lines was demonstrated. The self-assembled nanostructures of the peptide-drug conjugates can serve as reservoirs to release bioactive drugs in response to chemical and biological signals.

### Jigsaw-Shaped Self-assembling Peptide (JigSAP)

**Fig. 4:**
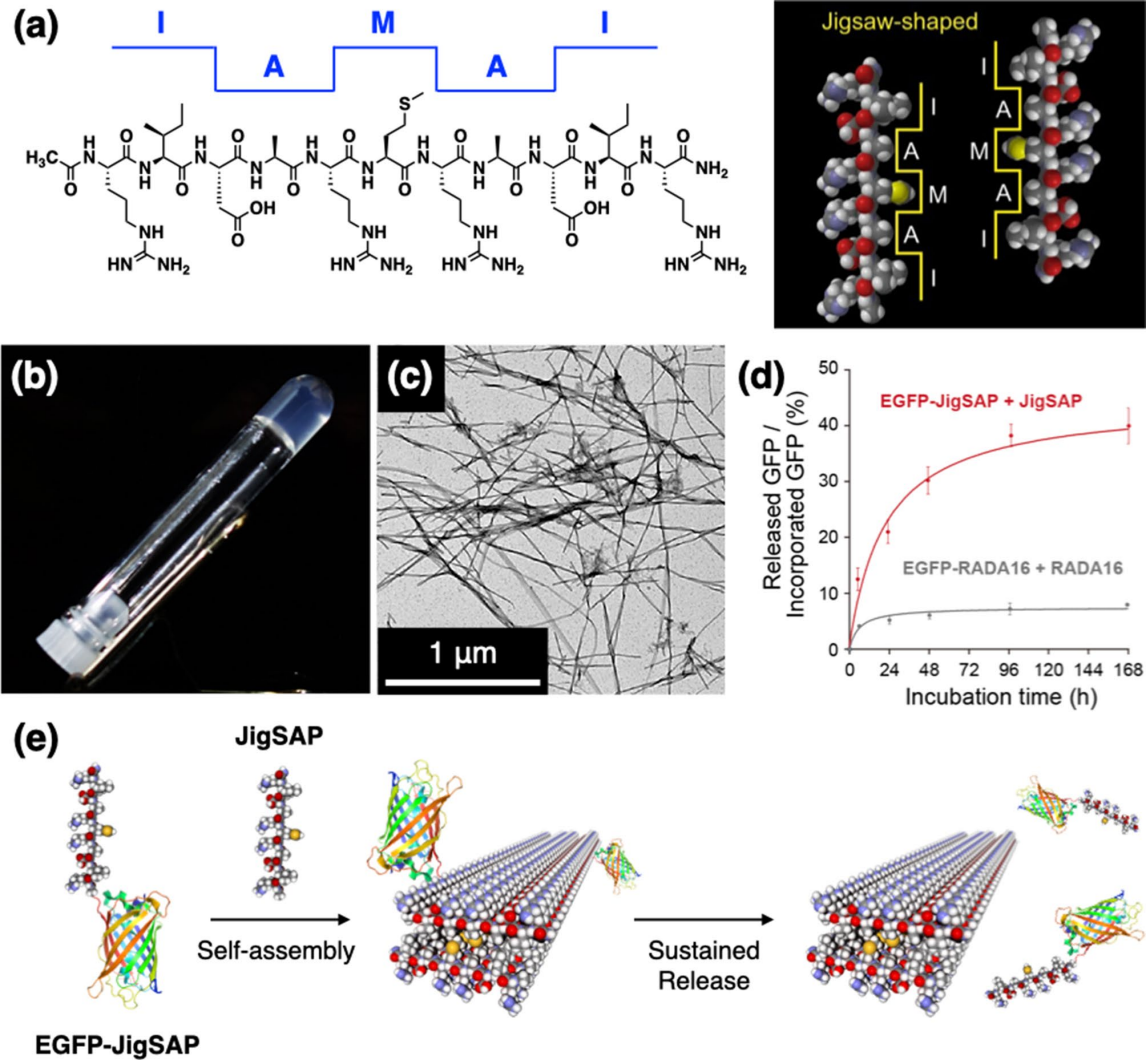
Self-assemblies and the efficient protein incorporation and release functions of JigSAP. **a** Molecular structure of JigSAP and schematic illustration of its self-assembly. **b** Photograph of a hydrogel of JigSAP in DMEM buffer at 37 °C (peptide concentration: 1.0 wt%, pH 7.4). **c** Transmission electron micrograph of JigSAP. Stain: uranium acetate. **d** The ratios of released EGFP to incorporated EGFP. EGFP-JigSAP (red) and EGFP-RADA16 (gray) were released from JigSAP and RADA16 peptide nanofibers, respectively. **e** Schematic illustration of incorporation of EGFP-JigSAP into a JigSAP supramolecular nanofiber and its sustained release

In the injured tissue regeneration process, secreted proteins bound with ECM are released to regulate various cellular events. In the case of severe tissue injury, however, both the secreted proteins and the ECMs are needed to be provided to promote efficient tissue regeneration because of the lack of suitable extracellular environments around the injured area. Although many artificial ECMs have been developed and used in clinical applications, the design of biocompatible materials that can incorporate and release secreted proteins remains unexplored. In 2021, Muraoka, Ajioka, and coworkers developed a jigsaw-shaped self-assembling peptide (JigSAP, Fig. 4a) [48]. This cell-adhesive fiber-forming self-assembling peptide can efficiently incorporate and release VEGF and demonstrate cell transplantation-free regenerative therapeutic effects at the subacute phase of ischemic stroke in mice. Inspired by the dovetail-packing motif of glycophorin A (GYPA) containing characteristic AXXXA sequence [49, 50], JigSAP consists of RIDARMRADIR. As the dovetail-packing motif of GYPA shows α-helix-to-β-strand structural transition, JigSAP transforms from a helical secondary structure to a β-sheet in an aqueous medium. JigSAP forms a hydrogel (Fig. 4b, c), and its stiffness (storage modulus) sharply enhances along with the conformational change. For supramolecular incorporation of full-length proteins into the JigSAP supramolecular nanofibers, the JigSAP tag was covalently attached to enhanced green fluorescence protein (EGFP) and VEGF (EGFP-JigSAP and VEGF-JigSAP). By incubating the peptide-tagged proteins with the excessive amount of the peptides, EGFP-JigSAP was incorporated into the nanofibers at the efficiency of 93 mol% in its active form. In comparison, the incorporation of non-tagged EGFP was only 3 mol%. Importantly, JigSAP hydrogel showed sustained release of the incorporated proteins bearing the JigSAP tag with higher efficiencies than conventional hydrogels such as RADA16 (Fig. 4d, e). VEGF-JigSAP was also incorporated in the JigSAP hydrogel and released efficiently. Sustained release of the JigSAP-tagged protein from the JigSAP hydrogel was also observed in vivo. The foreign body reaction level at the JigSAP injected area was comparable to the clinically commercialized RADA16 injected area. Seven days after ischemic brain stroke in mice, the JigSAP hydrogel incorporating VEGF-JigSAP was injected directly into the injured cerebral cortex. Seven days after the injection, behavioral recovery was improved based on a foot-fault test. Importantly, injection of JigSAP alone, VEGF-JigSAP alone, or JigSAP hydrogel incorporating VEGF without JigSAP tag hardly promoted the recovery. The injection of the JigSAP hydrogel incorporating VEGF-JigSAP enhanced angiogenesis and suppressed neuron death at the penumbra.

## Possible Mechanism of Injured Brain Regeneration at the Subacute Phase of Ischemic Stroke

As described above, VEGF replenishment is a key factor in promoting brain regeneration at the subacute phase of ischemic stroke. In 2000, Chopp and coworkers demonstrated VEGF effects on angiogenesis and neurological recovery by the continuous intravenous administration from 48 h after the onset of rat ischemic stroke, suggesting the importance of continuous VEGF replenishment for the recovery at the subacute phase [51]. Recent studies demonstrated the functional recovery by one-time injection of VEGF slow-release materials. Steinberg, George, and coworkers developed polyethylene glycol (PEG) polymer-based hydrogels delivering VEGF and MMP-9 and demonstrated the therapeutic effect [52]. Segura, Carmichael, and coworkers developed chemically cross-linked heparin nanoparticle hydrogels retaining VEGF and demonstrated the therapeutic effect of the subacute phase of ischemic stroke [53]. The study implicated the importance of neurogenesis and axon remodeling in addition to angiogenesis.

During CNS development, neuronal stem/progenitor cells give rise to newborn neurons, which migrate to the final destination and extend their neurites for proper development. These developmental events are also important for the recovery in the subacute phase of ischemic stroke (Table 1). Lindvall and coworkers found that the newborn neurons generated at the V-SVZ after stroke migrate into the damaged striatum area and differentiate into striatal spiny neurons [54]. Greenberg and coworkers generated Dcx-TK mice in which newborn and immature neurons are depleted after ganciclovir treatments and found that neurogenesis contributes to stroke recovery [55]. These studies demonstrated the critical role of adult neurogenesis from the postnatal V-SVZ in rodents. However, the existence of human adult neurogenesis in the hippocampus, the other area of adult neurogenesis, is still debated [56–59], while that of rodent adult neurogenesis is widely accepted. Future studies are required to solve whether adult neurogenesis in the hippocampus as well as the V-SVZ occurs or not. Thus, the strategy to enhance adult neurogenesis for the functional recovery of ischemic stroke may not apply to human therapy. In this sense, enhancing migration and axon guidance are reasonable strategies, as well as enhancing angiogenesis. Sawamoto and coworkers found that newborn neurons generated at the V-SVZ migrate toward the olfactory bulb in the tunnel enwrapped by the astrocytes during normal development [60]. Neuron-expressing Slit1 and astrocyte-expressing Robo2 play a key role in maintaining the astrocytic tunnel. After ischemic stroke, V-SVZ derived newborn neurons migrated toward the lesion under the tunnel enwrapped by reactive astrocytes in a Slit-Robo pathway [61]. When Slit1-overexpressing newborn neurons were transplanted, they migrated closer to the lesion and enhanced functional recovery. Axon guidance proteins also play a key role in functional recovery. Nogo and Nogo receptor (Nogo-NgR) pathway prevents axon growth and branching [62]. Strittmatter and coworkers implanted an osmotic pump releasing NgR-Fc, the fusion protein of the soluble ectodomain of NgR and the Fc domain of IgG, at the lateral ventricle to inhibit the Nogo-NgR pathway and found that NgR-Fc promoted functional recovery at the subacute phase of ischemic stroke [63]. Ephrin-A and EphA pathway controls the axon guidance of EphA-expressing neurons by suppressing axon sprouting [64]. Carmichael and coworkers injected EphA5-Fc-releasing hydrogels into the injured brain to inhibit EphA-signaling in neurons and found that EphA5-Fc promoted functional recovery of the subacute phase of ischemic stroke [65]. Thus, continuous replenishment of the key proteins important for neuronal development promotes injured brain regeneration.

**Table 1:** The list of proteins injected at the subacute phase and promoting the functional recovery of ischemic stroke models

Ischemic stroke model	Injection day after stroke	Delivering proteins	Materials or modification to release proteins	Injected area	Reference
Bilateral CCA occlusion (mouse)	7 days	VEGF and MMP9	8-arm PEG hydrogel	Into the injured cerebral cotex	[52]
Photothrombosis (mouse)	5 days	VEGF	Heparin nanoparticle	Into the injured cerebral cotex	[53]
dMCAO and photothrombosis (mouse)	7 days	VEGF	Self-assembling peptides (JigSAP)	Into the injured cerebral cotex	[48]
MCAO (mouse)	8 days	Slit1	Direct transplantation of the Slit1-overexpresisng V-SVZ cells	Into the injured striatum	[61]
MCAO (rat)	7 days	NgR-Fc	Osmotic minipump	Into the lateral ventricle	[63]
Photothrombosis (mouse)	7 days	EphA5-Fc	Hyaluronan and heparan sulfate proteoglycan hydrogel	Into the injured cerebral cotex	[65]

**Fig. 5:**
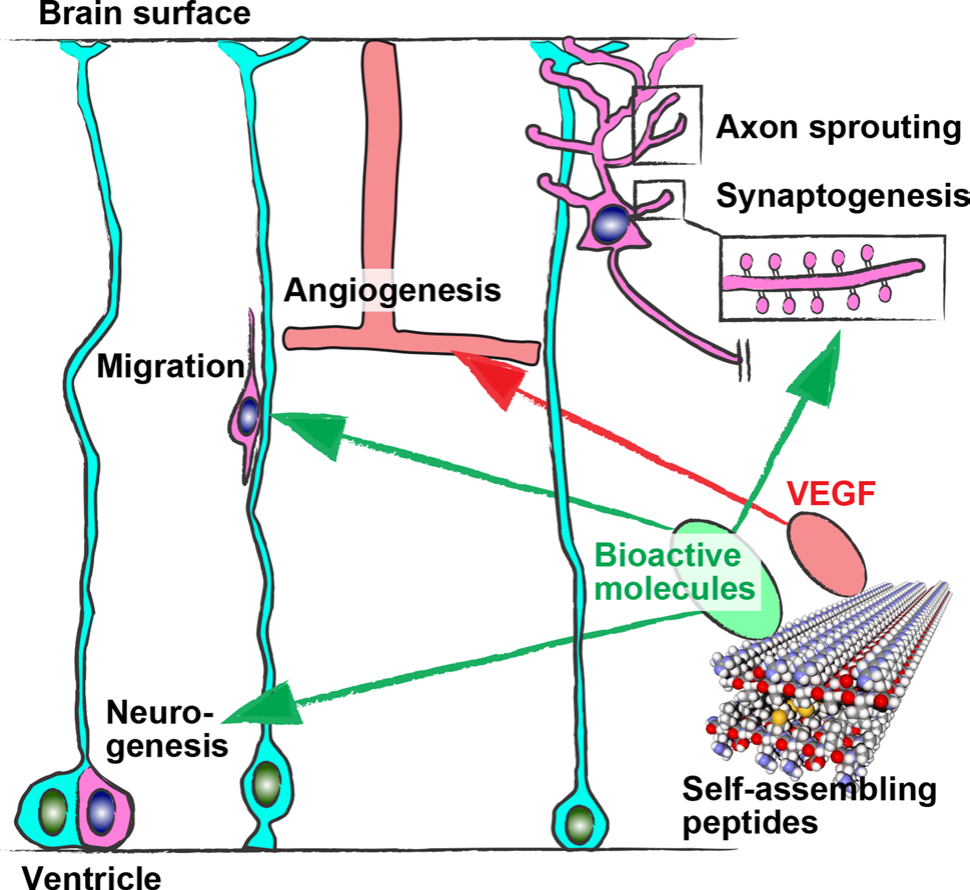
Developmental program activation by self-assembling peptides for injured CNS regeneration. During CNS development, neurons are generated from neuronal stem/progenitor cells. The newborn neurons migrate toward the final destination and undergo axon sprouting and synaptogenesis. Bioactive molecules incorporated into and released from self-assembling peptides can manipulate extracellular environments and activate such developmental programs, thereby promoting recovery after brain injury

Severe spinal cord injury causes irreversible disability because corticospinal axons do not regenerate. However, the transplantation of neuronal stem/progenitor cells enhanced the robust regeneration of corticospinal axons. Tuszynski, Poplawski, and coworkers found that the developmental transcriptome was activated in corticospinal neuron-enriched layer Vb neurons after spinal cord injury [66]. However, the developmental transcriptome was downregulated after two weeks, while that was still activated when corticospinal axons were regenerated by neuronal stem/progenitor cell transplantation. Thus, the developmental program activation by manipulating the extracellular environment is a rational strategy for injured CNS regeneration (Fig. 5).

## Conclusion

Although the mammalian brain is thought to have a limited regenerative ability after severe injury, activating the developmental programs during the subacute and regenerative phases enhances functional recovery. How can we activate such a developmental program? Transplantation of the cells activating such developmental programs is an attractive method in an experimental animal. Alternatively, the slow release of such developmental signals using chemically-crosslinked materials is also an attractive method in an experimental animal. However, there are potential difficulties in tuning the physicochemical properties and variances among lots for cell transplantation. Chemically-crosslinked materials also face the clinical application's difficulty because of the unidentified degraded products in vivo. In contrast, supramolecular peptides have an outstanding advantage for clinical application because of their unsurpassed functionalities, such as well-defined molecular and self-assembling structures enabling regulated biological, chemical, and physical properties and degradability into natural amino acids and peptides. Self-assembling molecular medicine may stand as a new drug modality of regenerative medicine in various tissues in addition to ischemic stroke.

## Data Availability

Enquiries about data availability should be directed to the authors.
